# Transcript and protein expression profile of PF11_0394, a *Plasmodium falciparum *protein expressed in salivary gland sporozoites

**DOI:** 10.1186/1475-2875-11-80

**Published:** 2012-03-23

**Authors:** Maggie S Schlarman, Renee N Roberts, Michael M Kariuki, Alexis N LaCrue, Ruguang Ou, Brenda T Beerntsen

**Affiliations:** 1 Department of Veterinary Pathobiology, University of Missouri, Columbia, MO, USA; 2 Molecular Microbiology and Immunology and Veterinary Pathobiology Joint Graduate Program, University of Missouri, Columbia, MO, USA; 3 Department of Global Health, University of South Florida, Tampa, FL, USA

**Keywords:** *Plasmodium falciparum*, Sporozoite, Gene expression, Protein expression

## Abstract

**Background:**

*Plasmodium falciparum *malaria is a significant problem around the world today, thus there is still a need for new control methods to be developed. Because the sporozoite displays dual infectivity for both the mosquito salivary glands and vertebrate host tissue, it is a good target for vaccine development.

**Methods:**

The *P. falciparum *gene, *PF11_0394*, was chosen as a candidate for study due to its potential role in the invasion of host tissues. This gene, which was selected using a data mining approach from PlasmoDB, is expressed both at the transcriptional and protein levels in sporozoites and likely encodes a putative surface protein. Using reverse transcription-polymerase chain reaction (RT-PCR) and green fluorescent protein (GFP)-trafficking studies, a transcript and protein expression profile of PF11_0394 was determined.

**Results:**

The PF11_0394 protein has orthologs in other *Plasmodium *species and Apicomplexans, but none outside of the group Apicomplexa. *PF11_0394 *transcript was found to be present during both the sporozoite and erythrocytic stages of the parasite life cycle, but no transcript was detected during axenic exoerythrocytic stages. Despite the presence of transcript throughout several life cycle stages, the PF11_0394 protein was only detected in salivary gland sporozoites.

**Conclusions:**

PF11_0394 appears to be a protein uniquely detected in salivary gland sporozoites. Even though a specific function of PF11_0394 has not been determined in *P. falciparum *biology, it could be another candidate for a new vaccine.

## Background

Malaria is still a major problem around the world due to the development of insecticide-resistant mosquitoes, drug-resistant *Plasmodium *parasites, and economic/political instability in areas of the world where malaria is a significant problem [1]. It is estimated that 225 million cases of malaria occur annually and, of those, 750,000 are fatal [2,3]. Because of these issues, it is critical for improved and/or new malaria control methods to be developed. The *Plasmodium falciparum *gene, *PF11_0394*, was chosen as a candidate for study due to its potential role in the invasion of host tissues based on an *in silico *data mining protocol. *PF11_0394 *was selected based on data collected from PlasmoDB, the *Plasmodium *database, indicating that this gene likely encodes a putative surface protein, and is present in the sporozoite both at the mRNA transcript and protein levels [4-7]. These specific criteria were set for two reasons: first, not many sporozoite proteins are well characterized based on the difficulty of working with *P. falciparum *mosquito stages in a laboratory setting and, second, many *Plasmodium *proteins that have been shown to be critical for invasion and/or development of the parasite are surface or secreted proteins, including the sporozoite proteins, circumsporozoite (CS) protein and thrombospondin-related anonymous protein (TRAP) [8-10].

This study characterized in depth the *P. falciparum *gene, *PF11_0394*, at the transcript and protein levels to determine its expression profile during various life cycle stages. Using reverse transcriptase-polymerase chain reaction (RT-PCR) and green fluorescent protein (GFP)-trafficking studies, it was determined that *PF11_0394 *has transcript present during several life cycle stages, but its protein is only detected during the salivary gland sporozoite stage.

## Methods

### Parasite maintenance, parasite transmission, and cell cultures

*Plasmodium falciparum *strain NF54 was used for the following experiments and was maintained according to procedures described by Carter and colleagues [11]. Briefly, *P*. *falciparum *cultures were maintained in human blood (O + male, BioChemed Services) at a 6% haematocrit in complete culture medium, RPMI 1640 (Gibco) supplemented with 25 mM HEPES (Gibco), 0.5% Albumax (Invitrogen), and 0.005% hypoxanthine (Sigma). The medium was changed every 48 hours and the parasites were kept in a gas mixture (3% O_2_, 3% CO_2_, and 94% N_2_) at 37°C [11]. Cultures were diluted/split to prevent the parasitaemia from becoming too high by addition of fresh, washed 50% blood (blood washed three times with sterile RPMI and diluted 50:50 with RPMI), maintaining a 6% haematocrit level. The use of human blood was in compliance with federal guidelines and institutional policies. All experiments described in this paper were approved by the Institutional Biosafety Committee (IBC), the Institutional Review Board (IRB), and the University of Missouri Institutional Animal Care and Use Committee (IACUC).

In order to obtain infected mosquitoes to study the parasite stages within the vector host, four-five day old, female *Anopheles stephensi *were exposed to *P. falciparum*-infected blood (1:1 ratio of infected blood and human serum), using induced gametocyte cultures. Gametocyte cultures were produced by setting a standard culture (described above) at a parasitaemia of between 0.5-1.0% and maintaining them in complete culture media supplemented with 10% human serum (A + male, Interstate Blood Bank); however, instead of splitting the parasites with fresh red blood cells (RBCs), the culture was left undiluted such that a high parasitaemia developed and the parasites became stressed. This was done for 16 days and resulted in a mixture of male and female gametocyte stages (I-V), with a majority of them being mature stages. The mosquitoes were fed the infected blood for approximately 30 minutes, using a 37°C water-jacketed membrane feeding system. After the blood feed, only colony cages where at least 75% of females fed were utilized and the infected mosquitoes were maintained in an incubator (Low Temperature Illuminated Incubator 818, Precision) at 26-27°C with 82-88% humidity.

To obtain exoerythrocytic stages, axenic cultures (producing liver stage parasites without hepatocytes) were utilized and were produced by following an online protocol by Kappe and colleagues [12]. In brief, approximately 5 × 10^4 ^salivary gland sporozoites were added to a well of a 48-well plate (Corning) and allowed to incubate in RPMI 1640 medium (Invitrogen) supplemented with 10% foetal bovine serum (Hyclone) and 500 units/ml and 500 μg/ml penicillin and streptomycin, respectively. These cultures were maintained in a 37°C incubator with 5% CO_2 _for 24 hours before collection for transcript expression studies [12].

### Mosquito maintenance

*Anopheles stephensi *were used for all studies. Mosquitoes were reared using protocols available from the Malaria Research and Reference Reagent Resource Center (MR4). In brief, larvae and adults were maintained in an insectary with 78-85% humidity at approximately 26-27°C on a 12-hour light/dark cycle. Larvae were fed both a mixture of 0.33 g yeast (Fleischmann's) and 0.66 g micron (Sera) per 50 ml water and game fish chow (Purina). Adults were fed sucrose (0.3 M) *ad libitum*.

### Selection of candidate gene, *PF11_0394*

An *in silico *data mining procedure was used to select *PF11_0394 *as a gene of interest. Briefly, PlasmoDB was utilized to search for *P. falciparum *proteins predicted to be expressed only by the sporozoite and containing a signal peptide, increasing its probability of being a surface protein [6,13]. Next, additional sequence analysis programs available on the ExPASy Bioinformatics Resource Portal and SoftBerry, such as PSORT and ProtComp, were used to verify that the proteins encoded by the genes were predicted to either be located on the surface and/or secreted by the parasite [14,15]. Those proteins that were verified by these two programs to meet the required criteria (and were not proteins that had been studied or were currently being studied) became the genes of interest, including *PF11_0394*.

### PF11_0394 sequence analysis

Using PlasmoDB, the full genomic DNA (gDNA), complimentary DNA (cDNA), and protein sequence of PF11_0394 were obtained. A list of orthologs of PF11_0394 was compiled using both PlasmoDB and the National Center for Biotechnology Information's (NCBI) BLAST analysis program [16]. The ortholog sequences were then aligned using Vector NTI (Explorer or Contig Express, Invitrogen). Additional sequence information for PF11_0394 was obtained by using software programs such as TargetP, SignalP, PSORT II, WoLF PSORT, and PROSITE, all found via the ExPASy Bioinformatics Resource Portal [14,15,17].

### Isolation of *Plasmodium falciparum*-infected tissues for transcript expression studies

#### Oocyst sporozoites

*Anopheles stephensi *were infected with *P. falciparum *as described previously. Ten days post-infection (PI), when oocyst sporozoites were mature using laboratory conditions, 50 mid-guts were dissected from the abdomens of *An. stephensi *that had fed on a *P. falciparum*-infected blood meal. The tissues were placed in 50 μl IX phosphate buffered saline (10X PBS, 0.2 M phosphate buffer and 1.5 M NaCl pH 7.0, diluted 1:10 with Millipore water) in microcentrifuge tubes, snap-frozen in liquid nitrogen, and stored at-80°CCutil needed for RNA isolation.

#### Salivary gland sporozoites

Fifty sets of *An. stephensi *salivary glands were dissected from mosquitoes that had fed on a *P. falciparum*-infected blood meal 14 days PI because sporozoites are found in the glands at this time under laboratory conditions. The tissues were put in 50 μl 1X PBS in microcentrifuge tubes, snap-frozen in liquid nitrogen, and stored at -80°C until needed for RNA isolation.

#### Exoerythrocytic stages

Axenic exoerythrocytic stages were generated as previously described. After 24 hours, Trizol (Invitrogen) was directly added to the cultures to begin the process of RNA isolation.

#### Mixed erythrocytic stages and gametocytes

*Plasmodium falciparum *cultures were maintained as previously described. Either mixed erythrocytic stage (ES) cultures (containing a mixture of rings, trophozoites, and schizonts) or 16-day-old mixed gametocyte cultures (containing a mixture of stage I-V gametocytes, but with more mature forms present) were collected by centrifugation at 2,650 × g for five minutes. The infected RBCs were lyzed with 0.05% saponin (Invitrogen) in complete culture medium for three minutes at room temperature (RT) and parasites collected by centrifugation for five minutes at 2,650 × g. Purified parasites were then washed once with RPMI 1640 medium and collected again by centrifugation as previously described. The parasite pellets were stored at -80°C until needed for RNA isolation.

### RNA/DNA isolation and transcriptional analysis by reverse transcription-polymerase chain reaction (RT-PCR)

Total RNA was isolated from *Plasmodium*-infected tissues using a Trizol reagent-based protocol, following the manufacturer's instructions (Invitrogen). The samples were all DNase-treated (Promega), according to the manufacturer's instructions, to remove any contaminating gDNA. Approximately 2-3 μg of the DNase-treated RNA was used to synthesize cDNA using OligoDT primers from a SuperScript™ III First-Strand Synthesis System (Invitrogen), following the manufacturer's instructions.

Genomic DNA was isolated following the manufacturer's instructions using a DNeasy^® ^Blood and Tissue Kit (Qiagen) and was used as a positive control for all RT-PCR experiments. *PF11_0394 *full-length gene specific primers (5'-atgaaaatttttaattacatatgtg-3' forward and 5'-ttatataatatttctattatcttcc-3' reverse) were used to amplify a 762 base pair (bp) gDNA fragment and a 561 bp cDNA fragment in a polymerase chain reaction (PCR) using 2.0 μl gDNA (~100 ng total)/cDNA (~1/10 the volume synthesized from above), 1.25 units GoTaq^® ^DNA Polymerase (Promega), 1X GoTaq^® ^Flexi Buffer, 1 mM MgCl_2_, 0.2 mM di-nucleotide tri-phosphate mix, and 0.5 μM primers. PCR conditions were as follows: an initial denaturing step of 95°C for three minutes, 35 repetitive cycles of denaturing at 95°C for 30 seconds, primer annealing at 56°C for 30 seconds and an extension at 62°C for three minutes, and then a final extension at 62°C for 10 minutes [18]. *Plasmodium falciparum heat shock protein-70 *was used as a positive control during the axenic exoerythrocytic stage studies since *PF11_0394 *transcript was not detected and the primers used were 5'-aggtatagaaactgtgggtgg-3' forward and 5'-gattggttggcatacagcttc-3' reverse. After PCR amplification, all samples were separated on a 1% agarose gel and stained with ethidium bromide (EtBr) for UV detection. The experiments using oocyst sporozoites and axenic exoerythrocytic stages were done in biological duplicates. The experiments with salivary gland sporozoites, mixed ES, and mixed gametocytes were done in biological triplicates.

### Creation of a PF11_0394/GFP-trafficking construct

To detect the presence of PF11_0394 protein throughout the life cycle of the parasite, a PF11_0394/GFP-trafficking construct was made by cloning base pairs 28-759 (excluding the stop codon) of the open reading frame of the gene into the pPM2GT vector (obtained from MR4) [19]. The primers used to amplify the region were 5'-ccgctcgagcgtcctttaagaaatggtg-3' forward and 5'-ccgcctaggtataatatttctattatcttcc-3' reverse. The restriction enzymes XhoI and AvrII (New England Biolabs) are underlined and were used for cloning into the pPM2GT vector. These primers were used to amplify a 732 bp product via PCR using 2.0 μl DNA (~100 ng total), 1.0 μl FastStart High Fidelity Taq Polymerase (5 U/μl Roche), 1X FastStart Buffer, 1 mM MgCl_2_, 0.2 mM di-nucleotide tri-phosphate mix, and 0.5 μM primers. The PCR was conducted using conditions previously described, but with an annealing temperature of 54°C [20].

The product was double-digested with XhoI and AvrII, along with the pPM2GT vector, separated via gel electrophoresis, gel-purified according to the manufacturer's instructions using QIAquick^® ^Gel Extraction Kit (Qiagen), and ligated with T4 DNA Ligase (Promega) following the manufacturer's instructions. Two microlitres of the ligation products were transformed into DH10B electrocompetant cells via electroporation and streaked on antibiotic resistant plates. Using colonies that grew on the plates, gDNA was isolated as previously described. The DNA was sequenced at the DNA Core Facility at the University of Missouri and aligned with the PF11_0394 sequence available from PlasmoDB using Vector NTI (Invitrogen) to confirm that the correct protein coding sequence was obtained.

### Transfection of parasites with the trafficking construct

Transfections of *P. falciparum *were carried out according to Crabb and colleagues [21]. Before performing the transfections, mixed ES parasite cultures were synchronized with 5% D-sorbitol (Sigma) for 10 minutes followed by two washes with RPMI 1640 (Gibco) at 1,600 × g for five minutes two days before transfection. In addition, plasmid DNA was isolated using a Plasmid Maxi Kit (Qiagen) and equilibrated in CytoMix (120 mM KCl, 0.15 mM CaCl_2_, 2 mM EGTA, 5 mM MgCl_2_, 10 mM K_2_HPO_4 _pH 7.6, and 25 mM HEPES pH 7.6). The synchronized *P. falciparum *ring stage NF54 parasites were electroporated (BTX 600, BTX Harvard Apparatus; 0.2 cm cuvette, 0.31 kV, 950 μF, maximum resistance) with 50 μg of the plasmid DNA in CytoMix [21]. Transfected *P. falciparum *cultures were maintained as previously described.

Two days following electroporation, media containing WR99210 (2.5 nM, Sigma) was added to the cultures to begin the process of selecting transfected parasites utilizing the human dihydrofolate reductase gene drug cassette present in all constructs [21]. To enrich for GFP-trafficking recombinants and eliminate episomal plasmids, parasites were subjected to at least three rounds of drug selection (three weeks on drug and three weeks off drug for each round).

To obtain a clonal population of parasites with no presence of wild-type parasites carrying episomes, a limiting dilution was performed on the transfected parasites. Parasites were seeded in 96-well plates (200 μl volume) at two concentrations, either 25% or 50% of wells would contain a single parasite, and maintained in a gassed modular incubator chamber (Billups-Rothenberg, Inc., 3% O_2_, 3% CO_2_, and 94% N_2_) at 37°C. Cultures were gassed every other day for 20 days. On days 7, 14, and 17, 0.4% fresh red blood cells were added. On day 20, 150 μl of the parasite culture were transferred to a 96-well plate to begin gDNA isolation for use in PCR and Southern blot analysis to determine if clonal populations of parasites had been successfully created. To isolate the gDNA, 50 μl of 6% saponin (Sigma) was added to the 150 μl of cultures in the 96-well plate and incubated for five minutes at RT. The plate was centrifuged for 15 minutes at 2,650 × g and supernatant removed. One hundred microlitres of 1X PBS was added to each well to wash the parasites and the plate was centrifuged as previously described. The 1X PBS was removed and 40 μl of down scale prep buffer (DSP, 1 M Tris-Cl pH 8.0, 1 M KCl, and 1 M MgCl_2_) working stock (985 μl DSP stock, 10 μl proteinase K, and 5 μl Tween 20) was added to each well and parasite pellets re-suspended in the DSP solution [22]. The plate was incubated for 30 minutes at 50°C and then for 10 minutes at 95°C. The resulting gDNA was stored at 4°C until further use. The remaining 50 μl of parasites were used for expansion and cryopreservation of promising clonal parasite populations.

### PCR and Southern blot verification of the GFP-trafficking construct

Integration of the transfected DNA at the correct location was verified for the PF11_0394/GFP clones by PCR and Southern blot analysis. To verify integration at the *PF11_0394 *locus by PCR, the primers 5'-atgaaaatttttaattacatatgtg-3' *PF11_0394 *gene specific forward primer and 5'-tccgtatgttgcatcacc-3' GFP reverse primer were used for the GFP-trafficking construct and 4.0 μl gDNA (isolated from the 96-well plates above), 1.25 units GoTaq^® ^DNA Polymerase (Promega), 1X GoTaq^® ^Flexi Buffer, 1 mM MgCl_2_, 0.2 mM di-nucleotide tri-phosphate mix, and 0.5 μM primers were used. PCR conditions were as follows: an initial denaturing step of 95°C for three minutes, 35 repetitive cycles of denaturing at 95°C for 30 seconds, primer annealing at 52°C for 30 seconds and an extension at 62°C for three minutes and 30 seconds, and then a final extension at 62°C for 10 minutes. The samples were all separated via gel electrophoresis (1% gel) and visualized via UV detection using EtBr.

Southern blotting was performed with gDNA isolated as previously described from ES parasites and DIG nonradioactive nucleic acid labelling technology (Roche) was used for visualization of the DNA. Genomic DNA (2-3 μg) that was digested with SapI and KpnI was hybridized with a 732 bp fragment of *PF11_0394 *created with the PCR DIG Probe Synthesis Kit following the manufacturer's instructions (Roche). Before hybridization, the DNA was separated on a 0.7% agarose gel and transferred to a positively charged nylon membrane (Osmonics) overnight via an upward transfer method. Following the manufacturer's instructions, the membrane was washed, hybridized with the above probes, and DNA products detected by autoradiography using the DIG Nucleic Acid Detection Kit (Roche).

### GFP-trafficking studies

The GFP-trafficking studies described below were done using two independent PF11_0394/GFP clones obtained via the limiting dilution process previously described. Each independent clone was used in a biological replicate, with a technical replicate conducted for each as well.

#### Mixed erythrocytic stages and gametocytes

Both *P. falciparum *mixed ES cultures and day 16 mixed gametocyte cultures were obtained by collecting 200 μl of infected blood from culture flaks. This protocol was followed for all experimental groups: PF11_0394/GFP, NF54 WT negative control parasites, and 3D7HT-GFP (obtained from MR4) positive control parasites [23]. The collected, infected blood was centrifuged for five minutes at 2,650 × g and the supernatant removed. The infected red blood cells (iRBCs) were re-suspended in 200 μl 1X PBS containing DAPI nuclear stain (1:1,000 dilution, Invitrogen) and incubated in the dark for five minutes at RT. The iRBCs were centrifuged again for five minutes at 2,650 × g, washed once with 200 μl IX PBS, centrifuged a final time for five minutes at 2,650 × g, and a small drop of the blood was placed on a slide. Coverslips were placed on the slides and they were viewed with a 100X objective using an Olympus BX51 inverted fluorescent microscope coupled with a X-Cite^® ^Series 120 fluorescent light source. The entire slide was scanned, with at least 100 iRBCs and 50 gametocytes observed for each group.

#### Zygotes and ookinetes

For all experimental groups listed above, six mid-guts were dissected from *P. falciparum*-infected *An. stephensi *24-30 hours PI. The mid-guts were placed in 1X PBS containing DAPI nuclear stain (1:1,000 dilution) and incubated at RT for five minutes. The mid-guts were then placed three per slide into 15 μl of Matrigel™ (BD Biosciences) and coverslips placed on top. The infected mid-guts were then viewed as described above. Due to limited numbers, at least nine zygotes and five ookinetes were observed for each group per replicate.

#### Oocyst sporozoites

For all experimental groups listed above, six mid-guts were dissected from *P. falciparum*-infected *An. stephensi *10 days PI. The mid-guts were placed in 1X PBS containing DAPI nuclear stain (1:1,000 dilution) and incubated at RT for five minutes. Three mid-guts per slide were then placed into 15 μl of 1X PBS and coverslips placed on the slides. The infected mid-guts were viewed as previously described. At least 75% of the mid-guts had infections with three-18 oocysts per mid-gut for each group.

#### Haemolymph sporozoites

Haemolymph sporozoites were collected by perfusing the body cavity of 10 *P. falciparum*-infected *An. stephensi *12 days PI with 1X PBS. Haemolymph for all experimental groups described above was collected in microcentrifuge tubes containing 40 μl 1X PBS with DAPI nuclear stain (1:1,000 dilution). The sporozoites were concentrated by centrifugation at 18,000 × g for five minutes, supernatant removed, and 10 μl of sporozoites spotted on slides containing 10 μl of Matrigel™ [17]. Coverslips were placed on top of the slides and they were viewed as described above. Due to the difficulty of isolating haemolymph sporozoites from *An. stephensi*, only three to five haemolymph sporozoites were observed for each group per replicate.

#### Salivary gland sporozoites

For all three experimental groups, six pairs of salivary glands were dissected from *P*. *falciparum*-infected *An. stephensi *13-20 days PI. The glands were placed in 1X PBS containing DAPI nuclear stain (1:1,000 dilution) and incubated at RT for five minutes. The glands were then placed into 15 μl of 1X PBS on a slide and coverslips placed on top. The infected salivary glands were viewed as previously described. For each experimental condition, at least 75% of the salivary glands were infected with hundreds of sporozoites observed per set of infected glands for each replicate.

## Results and discussion

### PF11_0394 sequence analysis

*PF11_0394 *is a 762 base pair (bp) gene on chromosome eleven, containing one intron, resulting in a 561 bp cDNA product. The cDNA product encodes a 186 amino acid *P. falciparum *protein with an estimated molecular weight of 21,026 Daltons. Initial PlasmoDB data, based upon mass spectrometry results and sequence analysis, suggested that the PF11_0394 protein was expressed by salivary gland sporozoites, has a signal anchor, and four transmembrane domains (amino acids 28-50, 65-84, 97-119, and 149-171) [7,24]. To confirm these data and obtain more information about PF11_0394, additional sequence analysis programs were utilized. SignalP revealed that PF11_0394 is predicted to have a non-cleavable signal anchor [25]. Analysis using TargetP predicted that the protein enters the secretory pathway and, more specifically, is predicted to be a plasma membrane protein (located on the surface of the parasite) according to PSORTII and WoLF PSORT [26,27]. Additional sequence analysis using PROSITE, PROTCOMP, Profam, and NCBI (BLASTp) sites predicted that the protein has no GPI-anchor, has multiple glycosylation and phosphorylation sites and has no functional identity with other known proteins [16,28].

**Figure 1:**
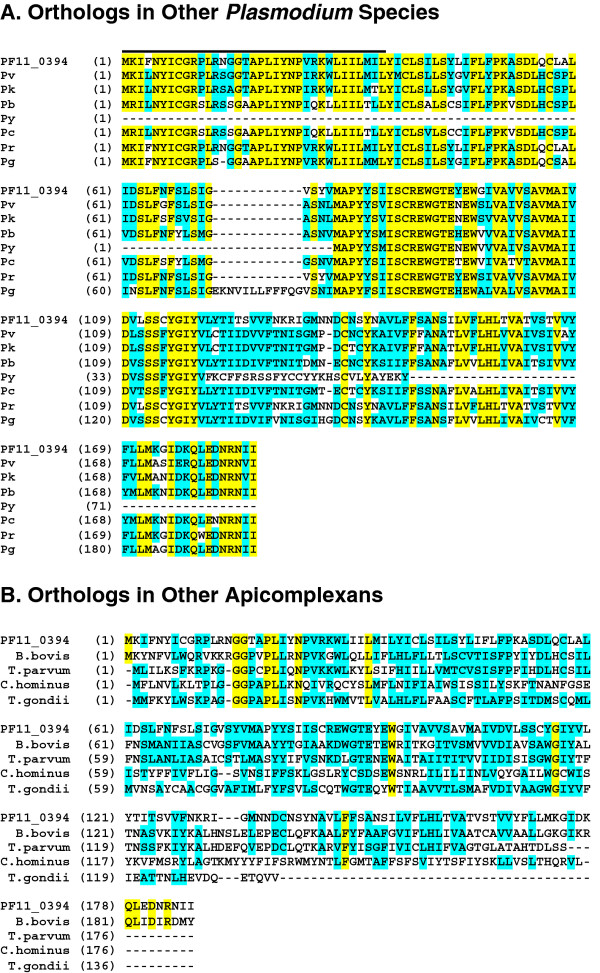
**PF11_0394 has orthologs in other *Plasmodium *species (A) and other Apicomplexans (B)**. BLAST analysis of PF11_0394 showed that the protein has orthologs in other *Plasmodium *species (A), as well as other Apicomplexans (B); however, PF11_0394 does not have orthologs with any proteins from members outside of the group Apicomplexa. Amino acids highlighted in yellow are identical in all proteins and amino acids highlighted in blue are similar in a majority of the proteins. The PF11_0394 protein has homology with proteins in *Plasmodium vivax *(Pv = PVX_092525 in PlasmoDB), *Plasmodium knowlesi *(Pk = PKH_093600 in PlasmoDB), *Plasmodium berghei *(Pb = PBANKA_091050 in PlasmoDB), *Plasmodium yoelii *(Py = PY06419 in PlasmoDB), *Plasmodium chabaudi *(Pc = CHAS_071180 in PlasmoDB), *Plasmodium reichenowi *(Pr = reich166f05 in Sanger), and *Plasmodium gallinaceum *(Pg = Pgal0546c06 in Sanger). The PF11_0394 protein also has orthologs with other Apicomplexans, including *Babesia bovis *(Bb = BOV_I000760 in GenBank), *Theilaria parva *(Tp = XP_765419 in GenBank), *Cryptosporidium hominus *(Ch = Chro.30131 in CryptoDB), and *Toxoplasma gondii *(Tg = 38.m02365 in ToxoDB).

Next, PlasmoDB and results from a BLAST analysis identified orthologs of PF11_0394 in other *Plasmodium *species (Figure 1A). The PF11_0394 protein has homology with proteins in *P. vivax *(Pv = PVX_092525 in PlasmoDB), *Plasmodium knowlesi *(Pk = PKH_093600 in PlasmoDB), *Plasmodium reichenowi (*Pr = c000130608.contig1 in Sanger), *Plasmodium berghei *(Pb = PBANKA_091050 in PlasmoDB), *Plasmodium yoelii *(Py = PY06419 in PlasmoDB), *Plasmodium chabaudi *(Pc = PCHAS_071180 in PlasmoDB), and *Plasmodium gallinaceum *(Pg = c000315856.contig1 in Sanger). The PF11_0394 protein also has orthologs with other Apicomplexans (Figure 1B), including *Babesia bovis *(Bb = BBOV_I000760 in GenBank), *Theilaria parva *(Tp = XP_765419 in GenBank), *Cryptosporidium hominus *(Ch = Chro.30131 in CryptoDB), and *Toxoplasma gondii *(Tg = 38.m02365 in ToxoDB). The PF11_0394 protein does not appear to have any orthologs with other proteins from members outside of the Apicomplexan group. PF11_0394 appears to be a highly conserved protein within the genus *Plasmodium *(79.6-98% conserved when PF11_0394 is compared to its orthologs) and is also conserved in several other Apicomplexans. Like PF11_0394, the *Plasmodium *proteins listed here are all predicted to have signal anchors (minus *P. yoelii*, whose sequence is not complete), enter the secretory pathway, and are predicted to be plasma membrane proteins. In addition, the proteins do not have any known function and/or identity with known proteins, but based on their conserved identity it is predicted that they would have similar functions in Apicomplexan biology. Since PF11_0394 does not have homology with any known human protein, it could be a good target candidate for a new vaccine.

### *PF11_0394 *transcript is present throughout a majority of the *Plasmodium falciparum *life cycle

**Figure 2:**
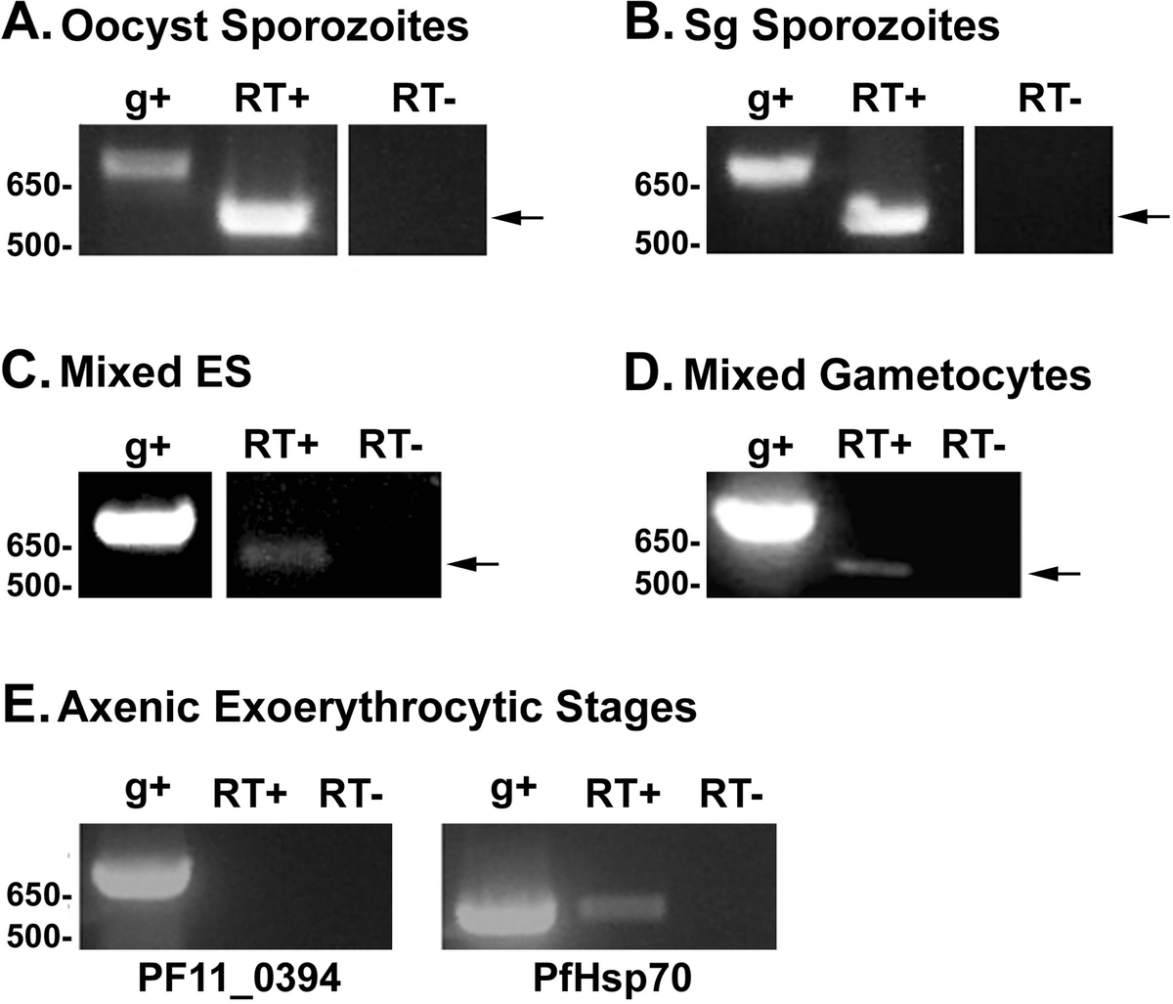
***PF11_0394 *transcript expression profile as shown by RT-PCR**. Primers specific for *PF11_0394 *were used to amplify cDNA fragments of the correct product size (RT+), indicating the presence of the transcript in oocyst sporozoites **(A)**, salivary gland sporozoites **(B)**, mixed erythrocytic stages (**C**, containing a mixture of rings, trophozoites, and schizonts), and mixed gametocyte stages (**D**, containing a mixture of stage I-V gametocytes). *PF11_0394 *transcript was not detected in axenic exoerythrocytic stages **(E)**; however, primers specific to the *Plasmodium falciparum heat shock protein-70 *(Hsp70) gene were used as a positive control to verify the presence of *P. falciparum *exoerythrocytic stages. Genomic DNA (g+) was used as a positive control and a no reverse-transcriptase (RT-) reaction was used as a negative control to show that the RNA was not contaminated with gDNA. *PF11_0394 *contains an intron, resulting in the size difference between gDNA and cDNA, and the arrows indicate the *PF11_0394 *RT-PCR products. This figure is a representative image of all the biological replicates conducted. Sg = salivary gland and ES = erythrocytic stages.

*PF11_0394 *transcript is present in oocyst sporozoites (Figure 2A), salivary gland sporozoites (Figure 2B), mixed erythrocytic stages (ES, cultures containing a mixture of rings, trophozoites, and schizonts) (Figure 2C), and mixed gametocyte stages (Figure 2D, cultures containing a mixture of stage I-V gametocytes), as demonstrated via RT-PCR (561 bp transcript). For all experiments, the gDNA positive control amplified the correct intron-containing product (762 bp) and the negative control (no reverse transcriptase) did not amplify any products.

Since generating exoerythrocytic stage parasites using primary human hepatocytes was not successful, a method to produce exoerythrocytic stages using axenic cultures (without the presence of liver cells) was utilized [12]. Data obtained using the axenic exoerythrocytic stages suggest that the *PF11_0394 *transcript is not present during exoerythrocytic stages when compared to an exoerythrocytic stage control gene, *P. falciparum heat shock protein-70 *(*PfHsp70*), which did amplify a transcript (Figure 2E). The *PfHsp70 *gene was used as a positive control since its transcript is highly up-regulated in exoerythrocytic stage parasites, but barely detectable in sporozoites [12]. This result indicated that exoerythrocytic stage parasites were indeed produced in the axenic cultures. Genomic DNA was used as a positive control and a no-reverse transcriptase reaction was used as a negative control as described above. Primers specific to the *P. falciparum circumsporozoite protein *gene (*PfCS*) were also used in the RT-PCR (data not shown). A transcript for *PfCS *was not amplified, even though the gene and protein have been shown to be expressed during the early exoerythrocytic stages [29]. This result demonstrates that even though axenic liver stage parasites were generated, as indicated by the presence of *PfHsp70 *transcript, they were likely produced at low levels. Therefore, the *PF11_0394 *transcript could potentially be detected if greater parasite numbers were present.

In addition to data available on PlasmoDB, a literature search was conducted for PF11_0394 and its predicted orthologs in two rodent malaria models, *P. berghei *and *P. yoelii*, to assess previous transcript and protein detection data generated throughout the life cycle of the parasite. According to the literature and PlasmoDB, *PF11_0394 *transcript is present during the salivary gland sporozoite stage and erythrocytic stages (specifically free merozoites, rings, trophozoites, schizonts, and gametocytes) [5,30-33]. Transcript results for exoerythrocytic stages vary between species, with a study in *P. falciparum *indicating no transcript expression and two studies, one using *P. yoelii *and one using *P. berghei*, confirming transcript expression [34-36]. Data obtained during these studies in *P. falciparum *for the salivary gland sporozoite stage, exoerythrocytic stages, mixed erythrocytic stages, and mixed gametocyte stages confirm results in the literature [5,30-36]. The presence of *PF11_0394 *transcript in oocyst sporozoites has not been described elsewhere and represents new knowledge about the *P. falciparum *transcriptome.

### The PF11_0394 protein is present in salivary gland sporozoites, as demonstrated by GFP-trafficking studies

Several attempts to produce PF11_0394 recombinant protein were employed, but were never successful. These included the use of several types of bacterial expression systems (Novagen), as well as a baculovirus expression system (Invitrogen). When using these systems, the PF11_0394 protein appeared to be expressed at low levels, but it could never be purified (even in the presence of protease inhibitors). Then, synthetic peptides were produced, using a commercial source (Genscript), and were used for IgY antibody production in chickens (Avian Immunology). Unfortunately, the peptides proved to be non-immunogenic, as the antibodies did not detect any proteins following Western blot analysis or produce any signals on immunofluorescent assays. PF11_0394 sequence analysis revealed that this protein has a signal anchor and four transmembrane domains, resulting in a largely hydrophobic protein embedded within the membrane of the parasite [37]. Thus, this feature of the PF11_0394 protein may have caused the protein production techniques used to be unsuccessful. Therefore, a PF11_0394/GFP-trafficking construct was created and used for protein trafficking studies.

**Figure 3:**
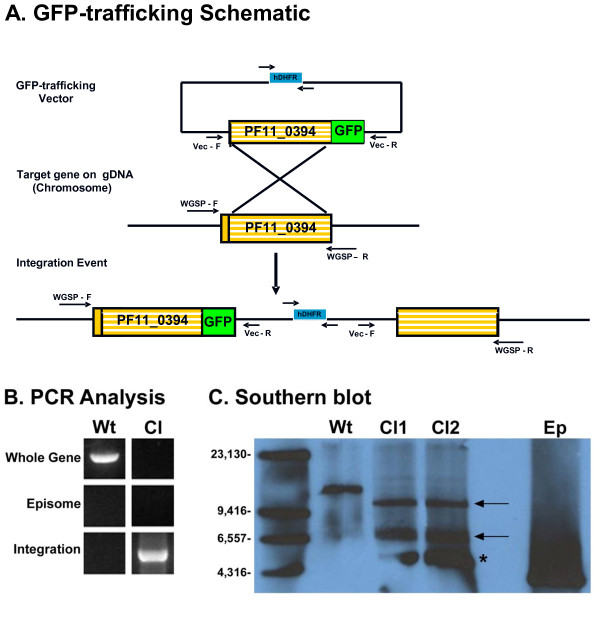
**Creation of two PF11_0394/GFP clonal parasite populations for protein trafficking studies**. **A**) Transfection schematic demonstrating how the PF11_0394/GFP construct was created using the pPM2GT vector (obtained from MR4) and successfully incorporated into the genome of the parasite via homologous recombination technology [19]. Expression of GFP is driven by the endogenous promoter of *PF11_0394*. Generation of two PF11_0394/GFP clonal parasite populations were verified by both PCR analysis (**B**) and Southern blot analysis using digoxigenin (DIG) technology coupled with autoradiography (**C**). Arrows indicate the predicted integration products of 10,454 base pairs and 6,602 base pairs using a PF11_0394 specific probe after restriction digestion with SapI and KpnI. The product indicated by an asterisk is of unknown origin and is likely a rearrangement of the plasmid that occurred after transfection. The drug cassette within the vector used for positive selection is human dihydrofolate reductase (hDHFR). GFP = Green fluorescent protein, Wt = Wild-type parasite genomic DNA, Cl = Clonal PF11_0494/GFP parasite genomic DNA, and Ep = PF11_0394/GFP plasmid DNA representing the episome. The arrows on the transfection schematic represent primers used for PCR analysis.

To assess the PF11_0394 protein detection profile throughout the life cycle of *P. falciparum *and confirm data found in the literature, a PF11_0394/GFP trafficking construct was created using the pPM2GT vector and transfected into the genome of the parasite via homologous recombination technology (Figure 3A) [19]. After a limiting dilution, two PF11_0394/GFP clones were isolated after initial PCR analysis (Figure 3B) confirmed that they had properly integrated into the genome of the parasite and the populations lacked the presence of wild-type (WT) parasites carrying episomes. These data were confirmed by Southern blot analysis (Figure 3C), as the predicted integration products of 10,454 base pairs and 6,602 base pairs were detected along with the absence of WT parasites carrying episomes. A Southern blot product of unknown origin was also detected and was likely caused by a rearrangement of the plasmid that occurred after transfection. This has been previously documented with the pPM2Gt vector [19].

**Figure 4:**
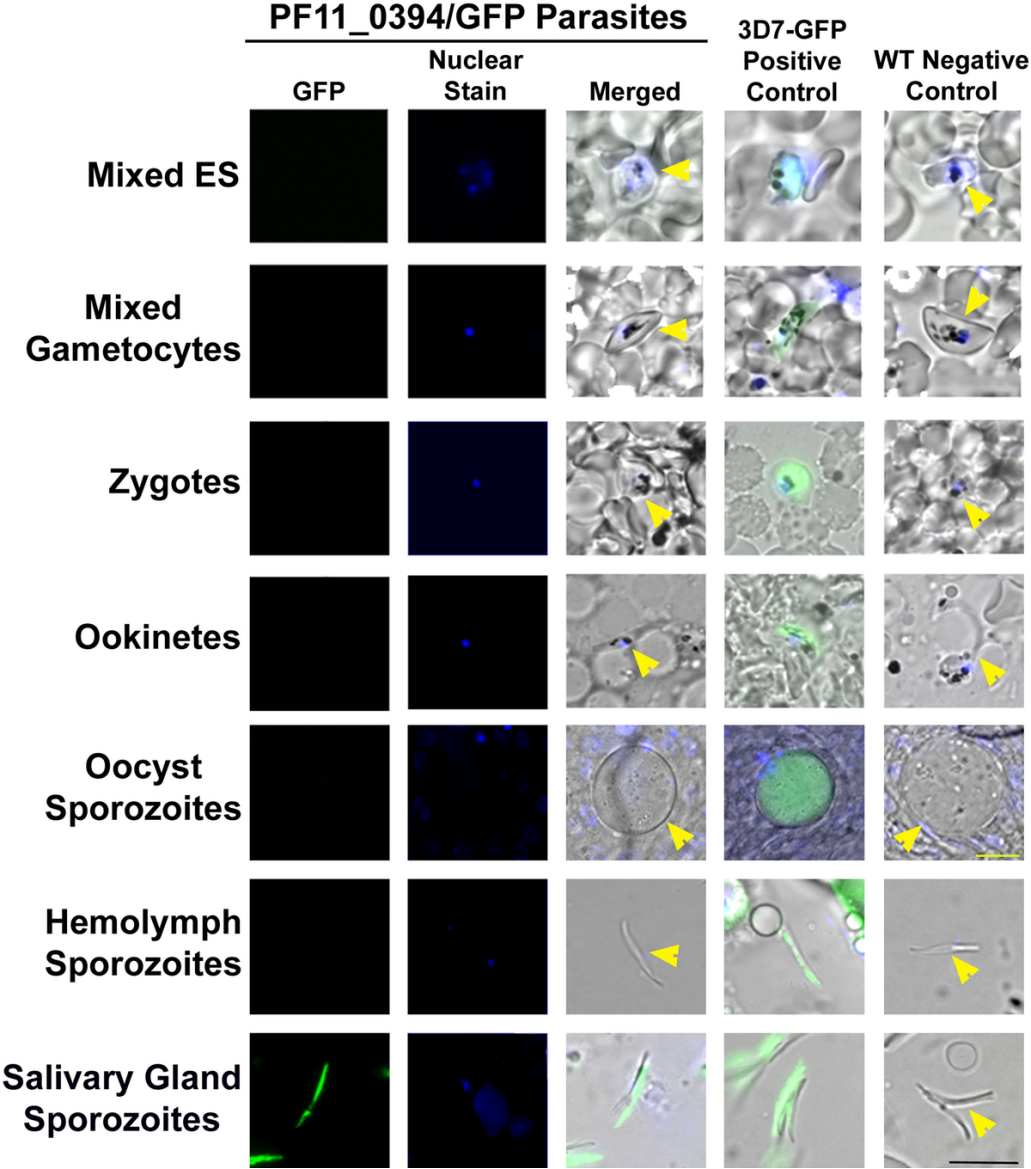
**PF11_0394/GFP-trafficking studies demonstrate that the PF11_0394 protein is present in salivary gland sporozoites**. The PF11_0394 protein is not detected during mixed erythrocytic stages (ES, culture representing a mixture of rings, trophozoites, and schizonts), mixed gametocytes (culture representing a mixture of stage I-V gametocytes), zygotes, ookinetes, oocyst sporozoites, and haemolymph sporozoites. The PF11_0394 protein is detected in salivary gland sporozoites. 3D7HT-GFP (3D7-GFP) constitutively expressing parasites were used as a positive control and wild-type (WT) parasites were used as a negative control [23]. This figure is a representative image from two biological replicates (one with each independent clone created) with two technical replicates each. Yellow arrowheads depict the presence of parasites that lack GFP expression (merged images). GFP = green fluorescent protein and nuclear stain = DAPI. The black scale bar in the lower right represents all of the images (except for the oocyst sporozoite stage) and is 10 μm. The yellow scale bar for the oocysts is 20 μm.

Using the two PF11_0394/GFP clones, two independent GFP-trafficking studies were completed (with two technical replicates each). WT parasites were used as a negative control and *P. falciparum *3D7HT-GFP parasites constitutively expressing GFP throughout the life cycle of the parasite were used as a positive control [23]. The various developmental stages of the parasite were examined using fluorescent microscopy (Figure 4). The stages observed were: mixed ES (contained a mixture of rings, trophozoites, and schizonts), mixed gametocytes (induced for 16 days and contained a mixture of stages I-V male and female gametocytes), zygotes (24 hours post infection, PI), ookinetes (24-30 hours PI), oocyst sporozoites (eight-10 days PI), haemolymph sporozoites (12 days PI), and salivary gland sporozoites (13-20 days PI). The PF11_0394 protein was not detected in mixed ES, mixed gametocyte stages, zygotes, ookinetes, oocyt sporozoites, and haemolymph sporozoites, despite a reasonable number of parasites being observed for each stage for each of the four replicates (see Methods). The PF11_0394 protein was only detected in salivary gland sporozoites (200/200 = 100% detection in salivary gland sporozoites counted, with hundreds of additional parasites observed as well). For all experiments, all of the 3D7HT-GFP parasites were positive for GFP expression and all of the WT parasites were negative (the parasites did not express GFP). These data confirm previous mass spectrometry results found by Florens and colleagues and Lasonder and colleagues that the PF11_0394 protein is present in salivary gland sporozoites and not in other life cycle stages they observed, which included gametes, salivary gland sporozoites, free merozoites, trophozoites, schizonts and gametocytes between the two groups [4,7]. These data also provide additional protein information for *P*. *falciparum *stages not previously studied, such as the zygotes, ookinetes, oocyst sporozoites, and haemolymph sporozoites.

Due to the difficulty of generating liver stage parasites *in vitro*, protein detection studies were not able to be conducted for PF11_0394 during this particular life cycle stage. Nevertheless, based on data suggesting transcript is not present during the exoerythrocytic stages, it is predicted that the PF11_0394 protein is not present during the liver stages; however, there is always the possibility that the PF11_0394 protein is expressed during the liver stages, especially early exoerythrocytic stage development.

### Attempts to generate a clonal population of mutant *PF11_0394 *parasites to assess a potential function of PF11_0394 were not successful

*PF11_0394 *gene disruption constructs were made for functional analysis by cloning base pairs 37-700 of *PF11_0394 *into both the pHD22y and pCAM-BSD vectors [15,38,39]. Unfortunately, once the constructs were transfected into *P. falciparum*, integration of the constructs was never observed via PCR analysis, even after four rounds of drug selection. The PF11_0394 protein is not expressed in mixed erythrocytic stage parasites (the stage of the life cycle when transfections are conducted), so the failure to obtain a mutant PF11_0394 parasite population was not likely due to it being essential during this life cycle stage. The lack of integration could be due to the genome of *P. falciparum *being highly AT-rich (~80%) with greater than 90% of it occurring in the intronic regions of the genome [40]. Because *PF11_0394 *has one intron, it is a possibility that when the *PF11_0394 *disruption constructs were transfected into the *P. falciparum *genome, the highly AT-rich intron of *PF11_0394 *may have caused the constructs to homologously recombine with either other AT-rich areas of the *P. falciparum *genome or perhaps, with another gene that has an intron of similar sequence.

## Conclusions

In summary, data obtained from these studies demonstrate that PF11_0394 is a *P. falciparum *protein that has orthologs in other *Plasmodium *species and also has orthologs with other Apicomplexans. PF11_0394 does not have orthologs with any protein outside of the Apicomplexan group and shares no functional identity with other known proteins. PF11_0394 is thus a novel protein to study in *P. falciparum *biology. Transcript detection studies determined that *PF11_0394 *transcript is present throughout a majority of the life cycle of the parasite, including mixed ES, mixed gametocyte stages, oocyst sporozoites, and salivary gland sporozoites, but is not detectable during axenic exoerythrocytic stages. Protein detection studies demonstrated that the PF11_0394 protein is present in salivary gland sporozoites and not in other stages examined.

Overall, these data obtained for PF11_0394 have determined that the PF11_0394 protein is present in salivary gland sporozoites and, therefore, may be required for parasite or sporozoite development/survival in the mosquito salivary glands and/or development within and/or invasion of human host tissues. A few *Plasmodium *sporozoite proteins have been found to be critical for development and/or invasion of host tissues. For example, both the CS protein and TRAP are sporozoite proteins that are essential for proper development and invasion of both mosquito salivary glands and human hepatocytes [9,10,41-45]. Thus, based on the protein detection profile of PF11_0394, it could be another candidate gene for a pre-erythrocytic stage vaccine since it does not share identity with any known human protein.

## Competing interests

The authors declare that they have no competing interests.

## Authors' contributions

MSS designed experiments, analysed data, and wrote the manuscript. RNR, MMK, and ANL assisted in experimental design and manuscript edits. RO helped with preparing experimental reagents and ordering supplies. BTB supervised the research experiments and helped with experimental design, data analysis, and manuscript edits. All authors read and approved the final manuscript before submission.
